# Understanding partitioning in two-phase systems with induced miscibility gaps—comparing SWIEET and QuEChERS

**DOI:** 10.1007/s00216-025-05913-0

**Published:** 2025-05-27

**Authors:** Nadja Kalinke, Markus Kramer, Tim Neumann, Christoph Körber, Carolin Huhn

**Affiliations:** 1 Institute of Physical and Theoretical Chemistry, Department of Chemistry, Eberhard Karls Universität Tübingen, Tübingen, Germany; 2 Institute of Organic Chemistry, Department of Chemistry, Eberhard Karls Universität Tübingen, Tübingen, Germany; 3 Merck KGaA, Darmstadt, Germany

**Keywords:** Salt-free extraction, Solvatochromic dyes, Kamlet-Taft parameters, Water content, Phase separation

## Abstract

Our salt-free SWIEET extraction method, recently developed, uses a mixture of acetonitrile and isopropanol as extraction solvents for extractions from aqueous solutions. Glucose was added to induce a phase separation between an aqueous and an organic phase, both of which contain water, acetonitrile, and isopropanol, but in different fractions. During method development, we observed that the amounts of isopropanol or glucose added greatly influenced analyte recoveries. This study aims to better understand the effects underlying especially the enhanced extraction of very polar and charged analytes. We varied the type and concentration of additives in SWIEET double extractions and investigated their effects on the recoveries of a broad range of analytes. Furthermore, the organic and aqueous phases resulting from the extractions were characterized with NMR, solvatochromic dyes, and Karl Fischer titration, to gain a deeper understanding of the effects of phase composition, the polarity and water content of the phases, and the phase ratio. Significant differences between QuEChERS and SWIEET were observed regarding the water content of the organic phase. In contrast, differences in phase composition between SWIEET double extraction steps were minor, and we discussed the high phase ratio of the organic to aqueous phase to be a major reason for the enhanced recoveries of polar analytes in the second extraction step.

**Graphical Abstract:**

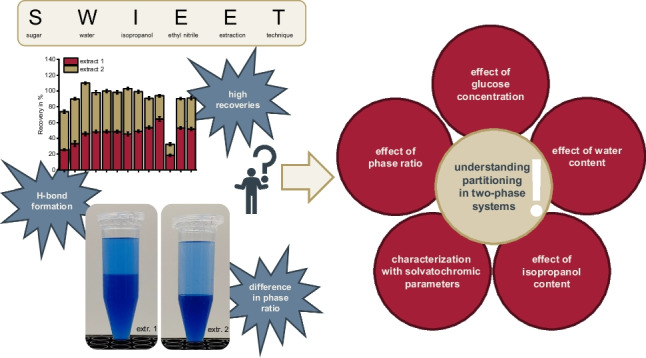

## Introduction

The efficiency of an extraction in liquid-liquid extraction is based on the analytes’ relative solubility in the donor and acceptor phases, and thus the distribution coefficient. Conventionally, nonpolar solvents are used to extract nonpolar analytes, and polar analytes are extracted with polar solvents [1], while mixtures can also be used to maximize solubility [2]. However, only a few methods evolved with a broad analyte coverage. Liquid-liquid extractions are based on the formation of a two-phase system, either through the use of two immiscible solvents or the induction of a miscibility gap, such as by sugaring-out in SWIEET (sugar water isopropanol ethyl nitrile extraction technique) [3] or salting out in QuEChERS extractions.

The SWIEET method uses sugars instead of salts to induce a phase separation in an acetonitrile-water mixture, with isopropanol as a protic solvent present, added to enhance recoveries. All method development steps, as well as the application of the SWIEET method to different sample types and a study of matrix effects, can be found in Kalinke et al. [3]. The optimized SWIEET method included two consecutive extraction steps to reach high recoveries, especially for polar analytes. The best average recoveries were reached when the organic mixture consisted of 20% isopropanol and with 2 M glucose added to the aqueous sample. Overall, higher recoveries were achieved with SWIEET than with QuEChERS in wastewater treatment plant effluent, with average recoveries of 69% compared to 66% with QuEChERS. Especially for polar analytes (logD < 0), recoveries were high in SWIEET extractions (70%) compared to QuEChERS (57%). In the first SWIEET extraction step, 37% were recovered on average for all analytes and 32% in the second. In comparison, recoveries from QuEChERS double extractions were already high in the first step (57%) while only 9% were additionally recovered in the second step [3].

The reason behind these differences between SWIEET and QuEChERS is further investigated in this work. The two phases resulting from the liquid-liquid extractions are never pure; the organic phase will always contain some water, and the aqueous phase will contain a certain fraction of the organic solvent(s). The polarity of the phases, therefore, differs from the polarity of the neat solvents, and the polarity may be tuned by the fractions of water and organic solvent. The water content in the QuEChERS organic phase is known to be low not only due to the addition of NaCl, but especially due to the addition of MgSO_4_, which is added to dry the organic phase [4]. This decreases the polarity of the organic phase. Thus, target analytes of medium to low polarity can be well extracted from an aqueous phase, and separation from polar matrix components, which remain in the aqueous phase, is well possible. The high fraction of organic solvents in the organic phase also ensures compatibility with further matrix removal by dispersive solid phase extraction. In the original QuEChERS publication [4], the water content in the organic phase was determined with NMR via the chemical shift of the proton signal of water, which depends on the amount of water in the sample. For example, the water content was up to 130 mg/mL in apple extracts. The rather low water content of the organic phase in QuEChERS limits the extraction efficiencies of polar and charged analytes. This was one of the main reasons to establish the new liquid-liquid extraction method SWIEET, which was designed to enhance the extraction of polar analytes while keeping high extraction efficiencies for analytes of medium to high polarity. A major aspect was the addition of the polar and protic solvent isopropanol to the extraction mixture, intended to enhance the solubilization of charged compounds via hydrogen bond formation. Salts were omitted and double extractions were conducted and shifted the phase equilibrium of analytes to result in overall higher recoveries.

In order to better understand the solubilization and enhanced extraction efficiencies of polar and charged analytes, this study intends to look at the physicochemical aspects of the extraction process. The polarity of solvents, and especially solvent mixtures, and the type and intensity of solvent-solute interactions can be studied via the use of solvatochromic dyes [5]. Solvatochromism describes the dependence of the absorption maximum of a probe molecule on solvent polarity and other aspects of solute-solvent interactions. This is based on the difference in stabilization of the ground or excited state of the probe molecule. If the molecule has a nonpolar ground state, the excited state will be better stabilized with increasing solvent polarity, decreasing the excitation energy. This is called a positive solvatochromism and can be observed by a red shift (bathochromic shift) of the absorption maximum. For negative solvatochromism, on the other hand, a polar ground state will be better stabilized with increasing solvent polarity, leading to an increased excitation energy and a blue shift (hypsochromic shift) of the absorption maximum [5]. Based on this phenomenon, various empirical parameter scales were developed using single or multiple solvatochromic probes to describe solvent polarity [5]. The most popular approaches are the E_T_(30) scale and the Kamlet-Taft parameters, described below.

### Reichardt’s dye—the E_T_(30) parameter

The E_T_(30) parameter is based on the absorption maximum of the betaine dye “Reichardt’s dye”. This molecule was designed as a solvatochromic probe to cover a broad range of polarizability and to have an absorption in the visible range of 453–810 nm. The molecule’s ground state is better stabilized in environments of increasing solvent polarity, compared to the less dipolar excited state [6]. This results in a higher excitation energy in polar solvents and therefore a negative solvatochromism.

The empirical E_T_(30) parameter is based on the electronic excitation energies of Reichardt’s dye in the solvent of interest and can simply be calculated by converting the absorption maximum obtained from photometric detection in the solvent of interest to kcal/mol, according to Eq. (1) [5] from the wavelength at the absorption maximum λ_max_, a constant combining Planck’s constant *h*, the frequency at the absorption maximum *ν*_max_, the propagation of light *c*, and the Avogadro number N_A_:

1 $${\mathrm{E}}_{\mathrm{T}}30 \left(\frac{\mathrm{kcal}}{\mathrm{mol}}\right)=hc{\nu }_{\mathrm{max}}{N}_{A}=\frac{\mathrm{28,591}}{{\lambda }_{\mathrm{max}}}$$

E_T_(30) values range from 63.1 kcal/mol in water to 30.7 kcal/mol in tetramethylsilane. According to these values, solvents can be classified as protic (47–63 kcal/mol), aprotic (40–47 kcal/mol), or apolar (30–40 kcal/mol) [5].

### Kamlet-Taft parameters

Kamlet and Taft developed the parameters *α*, *β* and π* that can be derived from UV/Vis spectra of solvatochromic probes in different solvents. The parameter *α* is a measure of the hydrogen bond–donating capabilities of a solvent [7] and *β* for the hydrogen bond–accepting capabilities [8]. The parameter π* is used to describe solvent polarity-polarizability [9]. Kamlet-Taft parameters were determined in the literature for a large number of solvents and are often used in combination with a linear free energy relationship to compare solvent properties.

The method by González-Arjona et al. [10] was used for the determination of the Kamlet-Taft parameters *α*, *β*, and π* in this work. The absorption maxima of Reichardt’s dye E_T_(30), p-nitroanisole, and p-nitrophenol in the solvent mixtures of interest were converted to the unit kilokaiser (kK, 1000 cm^−1^) and used in Eqs. (2) and (3). For the determination of the polarity, 4-nitroanisole (NA) is used since it is not hydrogen bond accepting but is sensitive to dipolar interactions with the solvent. 4-Nitrophenol (NP) is a good hydrogen bond donor and can therefore assist in determining the hydrogen bond–accepting capabilities of a solvent.

2 $$\alpha =\frac{{\upnu }_{\mathrm{max}}\left({\mathrm{E}}_{\mathrm{T}}30\right)+1.873\cdot{\upnu }_{\mathrm{max}}\left(NA\right)-74.58}{6.24}$$

3 $$\beta =\frac{0.901\cdot {\upnu }_{\mathrm{max}}\left(NA\right)-{\upnu }_{\mathrm{max}}\left(NP\right)+4.16}{2.31}$$

The parameter π* was calculated from the absorption maximum (*ν*_max_) of 4-nitroanisole in the organic phases of the extraction method according to Eq. (4), with the literature values for 4-nitroanisole in cyclohexane *ν*_0_ = 34.12 kK and the empirical scaling factor *s* = −2.343 kK [10]. The π* values range from 0 for cyclohexane to 1 for dimethyl sulfoxide.

4 $${\uppi }^{*}=\frac{{\upnu }_{\mathrm{max}}-{\nu }_{0}}{s}$$

### Application of solvatochromic parameters

E_T_(30) parameters are widely applied, e.g., for the characterization of surfaces [11, 12] and ionic liquids [13, 14], but also for the characterization of mobile phases in liquid chromatography, as well as for the determination of the water content in organic solvents [6].

Solvatochromic parameters are often used in chromatography to understand retention through solute-solvent interactions [15]. Kamlet et al. [16] correlated solvation parameters to chromatographic capacity factors for a C18 stationary phase. For common eluents like acetonitrile/water and methanol/water, retention models based on Kamlet-Taft parameters were established [17]. Barbosa et al. [18] used such a model to select the optimal eluent composition for the separation of quinolones.

As intended in this work, solvatochromic parameters can be used to improve the understanding of extractions. Duchemin et al. [19] determined E_T_(30) values to determine changes in the extraction efficiencies for cesium from aqueous solution by the addition of alcohol modifiers to a crown ether solution. Bednarz et al. [20] investigated the E_T_(30) and Kamlet-Taft parameters of binary two-phase systems made of deep eutectic solvents, which were used for the extraction of phenolic compounds. Tang et al. [21] derived Kamlet-Taft parameters for aqueous biphasic systems and correlated them to the yield of paeonol extracted from a herb in a linear solvation energy relationship to optimize the extraction. Deng et al. [22] used Kamlet-Taft parameters of deep eutectic solvents to understand the extraction mechanism of sulforaphane from broccoli.

The parameter E_T_(30) was often used to characterize binary mixtures, as extensively reviewed by Spange [23]. However, describing the polarity of a binary solvent mixture remains complex, especially due to preferential solvation, which results in non-linear correlations of the E_T_(30) parameter and the composition of the mixture. Many theoretical approaches have been chosen to understand and prove preferential solvation of solvatochromic probes in binary mixtures [23]. Ternary mixtures have also been investigated in the literature, for example, by Jonquières et al. [24], who calculated E_T_(30) for a ternary alcohol/ether/polyurethaneimide system. Similarly, the E_T_(30) values of a methanol/acetonitrile/propanol system were investigated by Leitão et al. [25]. Kamlet-Taft and E_T_(30) parameters were calculated by Fletcher and Pandey [26] for an ionic liquid/ethanol/water mixture. Here, too, a non-linear correlation between the parameters and the composition of the mixture was observed. Nunes et al. [27] determined Kamlet-Taft and E_T_(30) values for methanol/formamide/acetonitrile mixtures, corroborating studies with other binary mixture solvation models. It should be recognized that the solvation mechanisms are not fully understood yet, and E_T_(30) values of mixtures should be interpreted carefully [23].

In this work, QuEChERS is used for comparison, since it is a liquid-liquid extraction technique using salts to induce a phase separation in an acetonitrile-water system, which is similar to SWIEET, which uses sugars. The aim of this study was to examine different SWIEET extraction media and determine phase composition and solvatochromic parameters of SWIEET and QuEChERS organic phases to gain understanding of the physicochemical aspects that affect partitioning and analyte extraction in two-phase systems.

## Materials and methods

### Chemicals

1-Ethyl-3-methyl-imidazolium (EMI, ≥95%), 2-methyl-4-chlorophenoxyacetic acid (MCPA), 5-amino-2-naphthalene sulfonic acid (ANSA, ≥95%), acesulfame (ACE, ≥99%), acetonitrile-d3 (>99.8%), acridine (ACR, 97%), alpha-d-glucose (96%), clarithromycin (CLA, ≥98%), dichloromethane (DCM, 99.9%), diclofenac sodium salt (DIC, ≥98 %), hexane (≥97%), isopropanol (iPr, LC-MS grade), maleic acid (>99%), methanol (MeOH, LC-MS grade), naphazoline (NAPHA, ≥98%), pindolol (PIN, 98%), saccharine (SAC, ≥98%), and toluene (99.9%) were purchased from Sigma-Aldrich (Steinheim, Germany). 4-Hydroxybenzoic acid (HBA, ≥98%) and *N*-diethyl-m-toluamide (DEET, ≥98%) were from Fluka (Buchs, Switzerland). 4-Nitroanisole (4-NA, >99%), ethyl acetate (EtOAc, p.a.), and water (LC-MS grade) were provided by Thermo Fisher (Kandel, Germany). 4-Nitrophenol (4-NP, ≥99%), acetonitrile (MeCN, LC-MS grade), dimethyl sulfoxide (DMSO, 99.5%), formic acid (>99%), and indigo carmine were bought from Roth (Karlsruhe, Germany). Metformin (MET, 97%) was from Alfa Aesar (Haverhill, MA, USA). Reichardt’s dye (RD) was purchased from Biosynth (Leicester, UK). Deuterium oxide (D_2_O, 99.9%) was from deutero (Kastellaun, Germany). Purified water, produced using a PURELAB Classic PL5241 (ELGA LabWater, Celle, Germany), was used throughout the study.

### Extraction procedure

For SWIEET extractions, water was spiked with an analyte mix of 13 model analytes to a final concentration of 3 mg/L each for the determination of recoveries. Glucose was added to a final concentration of 1.5, 2, or 2.5 M. For liquid-liquid extractions, 2.5 mL of this analyte mix was diluted with the same volume of an organic extraction mixture consisting of 80 or 90 vol.% acetonitrile and 20 or 10 vol.% isopropanol. The mixture was homogenized for 1 min using a vortexer. After a clear phase boundary was visible, usually within a minute, phases were separated by pipetting. For double extraction, 2.5 mL of fresh organic extraction mixture consisting of 80 or 90 vol.% acetonitrile and 20 or 10 vol.% isopropanol was added to the residual aqueous phase from the first extraction step. After mixing for 1 min using a vortexer, the phases were allowed to separate again.

QuEChERS extractions were conducted as described by Kalinke et al. [3]. Briefly, 2.5 mL acetonitrile and 0.25 mg NaCl and 1 mg MgSO_4_ were added to 2.5 mL water with the analyte mix to induce phase separation. For a modified QuEChERS extraction with isopropanol, an 80–20 acetonitrile-isopropanol mixture was used instead of acetonitrile.

To improve the visibility of the phase boundary and estimate the water content, indigo carmine, which is only soluble in water or in water-rich mixed phases, was added to the aqueous phase for some experiments.

The organic extracts were analyzed using a gradient elution RPLC coupled to a Q-ToF-MS. Details on the model analyte mix, RPLC-MS method, and quantification can be found in Kalinke et al. [3]. Recoveries were determined separately for the two extraction steps, and data are reported for single analytes or average values for all 13 analytes.

### Determination of solvatochromic parameters using UV-Vis spectroscopy

UV-Vis spectra of the dyes 4-nitroanisole, 4-nitrophenol, and Reichardt’s dye in various solvents were recorded using a Lambda 19 UV/Vis spectrometer (PerkinElmer, Waltham, MA, USA) with LambdaSPX software (ascansis OHG, Überlingen, Germany). The spectra were recorded from 170 to 900 nm in 1-nm intervals at a scan rate of 480 nm/min, with the blank extracts as the reference. The dyes were added to the separated phases after extraction without spiking the analyte mix, so that no bias was introduced due to partitioning.

### Determination of the water, isopropanol, and glucose content

For NMR analysis, 300 µL of the organic phase was combined with 200 µL of acetonitrile-d3. For the analysis of the aqueous phase, D_2_O was used. To quantify isopropanol, 5 mg of maleic acid were added to the sample. Four replicates of each organic and aqueous phase were measured, and integrals were averaged. The ^1^H spectra were recorded with a Bruker Avance III HD 400 at a frequency of 400 MHz and evaluated using Topspin 4.1.4 (Bruker, MA, USA). The spectra were calibrated to the acetonitrile signal at 1.94 ppm. The amount of isopropanol was calculated by relating the integrals of maleic acid (*I*_MA_, 6.26 ppm, s) and isopropanol (*I*_iPr_, 3.87 ppm, sept), considering the known amount of maleic acid (*n*_MA_) and the number of protons causing the isopropanol signal (*p*_iPr_=6) according to Eq. (5):

5 $${n}_{\mathrm{iPr}}=\frac{{n}_{\mathrm{MA}}}{{I}_{\mathrm{MA}}}\cdot \frac{{I}_{\mathrm{iPr}}}{{p}_{\mathrm{iPr}}}$$

In addition, the water content was determined by Karl Fischer titration at the analytical facilities at Merck, Darmstadt, Germany.

Glucose concentrations were determined by drying the organic and aqueous phases from SWIEET extractions conducted as described in “Extraction procedure” section at 70 °C, so that the amount of glucose can be determined by weighing. For this experiment, extraction volumes were reduced by a factor of 5 to shorten the drying time.

## Results

To better understand the reasons for the improved recoveries from SWIEET double extractions compared to QuEChERS and differences between the two SWIEET extraction steps, we first investigated the different SWIEET additive compositions regarding their ability to extract the model analytes as well as the resulting phase ratio. For the most noticeable phase composition, we determined water and isopropanol content, as well as solvatochromic parameters to gain insight into the phase compositions.

### Influence of glucose and isopropanol content on analyte recoveries

During the optimization of the SWIEET extraction method [3], we observed that the volume of the organic phase increased significantly after the addition of the fresh organic mixture (a mixture of 20% isopropanol and 80% acetonitrile) in the second extraction step. The composition of the aqueous phase after the first extraction step is unknown: Due to the double extraction and the addition of isopropanol, the increased glucose concentration is no longer simply correlated with increased recoveries, as described in the literature [28–30]. To further investigate the roles of glucose concentration, isopropanol content, and phase ratios on recoveries, further experiments with glucose concentrations in the aqueous sample ranging from 1.5 to 2.5 M and 10 or 20% isopropanol in the organic mixture were conducted. The ranges were chosen during preliminary experiments, which showed that lower glucose concentrations do not induce a stable phase separation, and higher glucose concentrations are above the solubility limit of 2.6 M in water. For the combination of 20% isopropanol content and a glucose concentration of 1.5 M, no phase separation was induced.

**Fig. 1:**
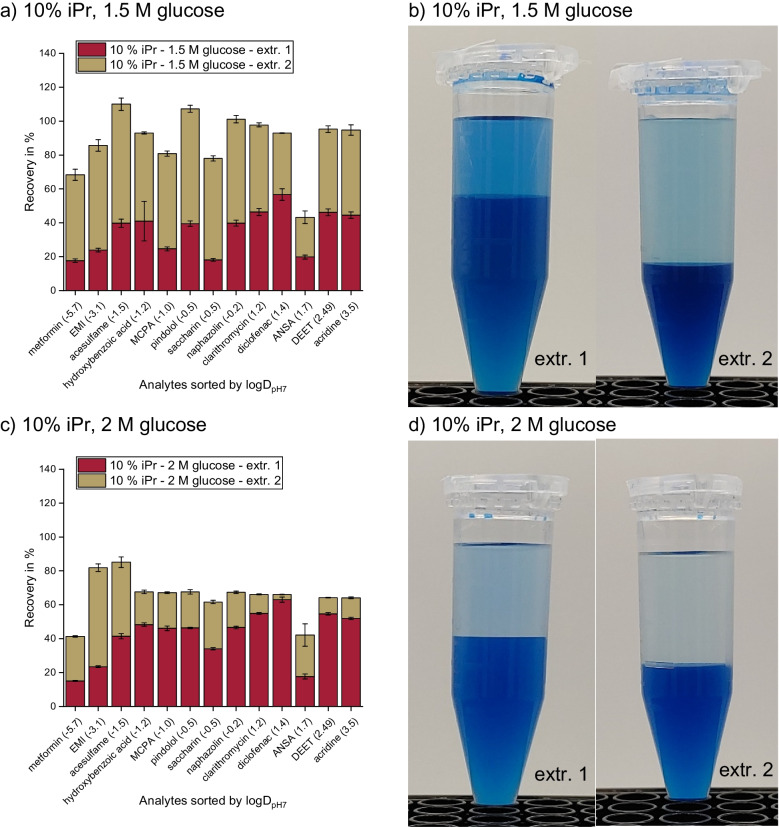

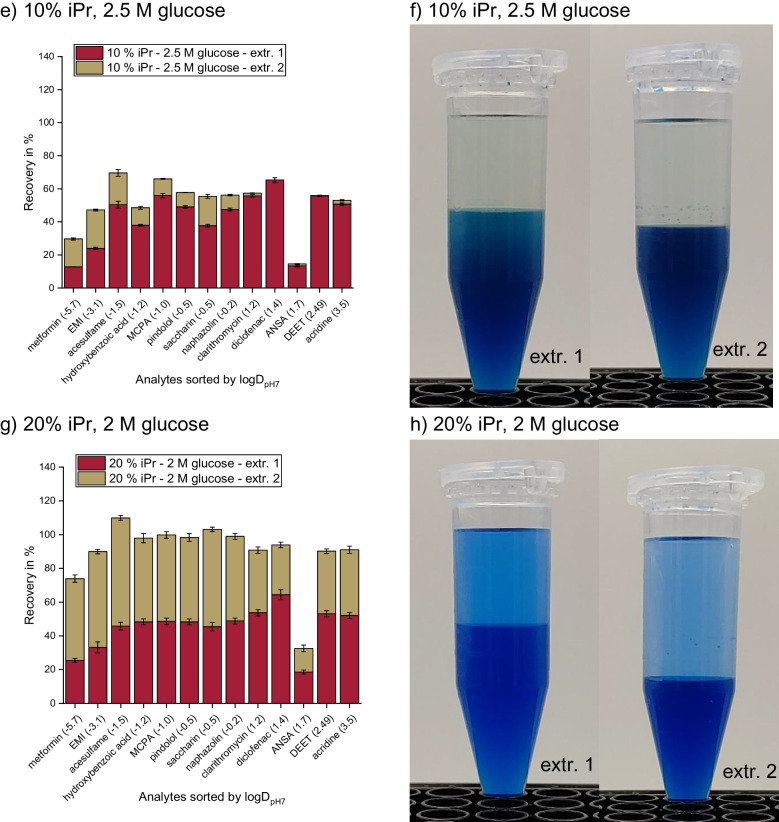

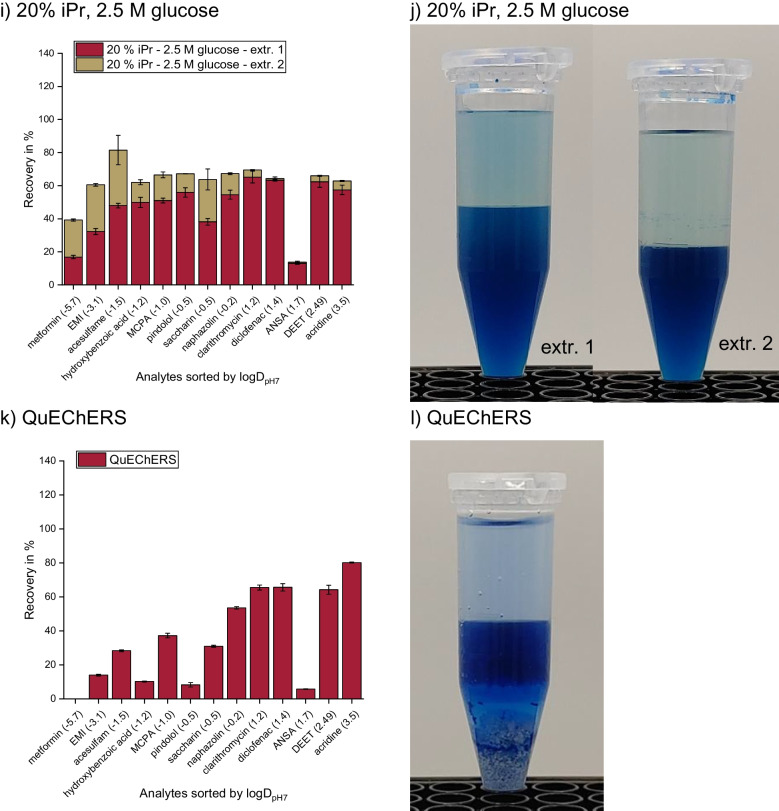
**A**–**J** Recoveries of SWIEET double extractions from water with varying isopropanol content in the organic mixture (10–20%) and glucose concentration in the aqueous mixture (1.5–2.5 M) (**a**, **c**, **e**, **g**, **i)** and photographs of the extraction vials after phase separation of extraction steps 1 (left) and 2 (right). **k**, Recoveries of QuEChERS extractions with the solid phase from excess salts visible. Phases were colored with indigo carmine (**b**, **d**, **f**, **h**, **j**, **l)** as a marker for the water content [3]. For all protocols, see “Extraction procedure” section

Considering first only the first extraction step with 10% isopropanol in the extraction mixture, we observed a slight increase in the average recovery from 35 to 42% when increasing the glucose concentration from 1.5 to 2 M (Fig. 1a vs. c), but no further improvement at 2.5 M (43%, Fig. 1e). The results for 2 M and 2.5 M glucose with 20% isopropanol added (45% and 47%, Fig. 1g and i) are similar to those at 10%, indicating a high robustness with regard to the isopropanol content in the first step. No combination tested significantly increased the recovery of a specific analyte or analyte class such as polar vs. nonpolar analytes in the first step.

For the second step, an increased sugar concentration in the aqueous sample led to decreasing recoveries: At 10% isopropanol content in the organic mixture, average recoveries decreased from 53 to 23 to 9% with increasing glucose concentration, and at 20% isopropanol, from 45 to 9%. Average recoveries were similar for 1.5 M glucose at 10% isopropanol and 2 M glucose at 20% (44% and 45%) in the second step. The same held true for 2 M glucose at 10% isopropanol and 2.5 M glucose at 20% isopropanol (33% and 31%).

**Fig. 2:**
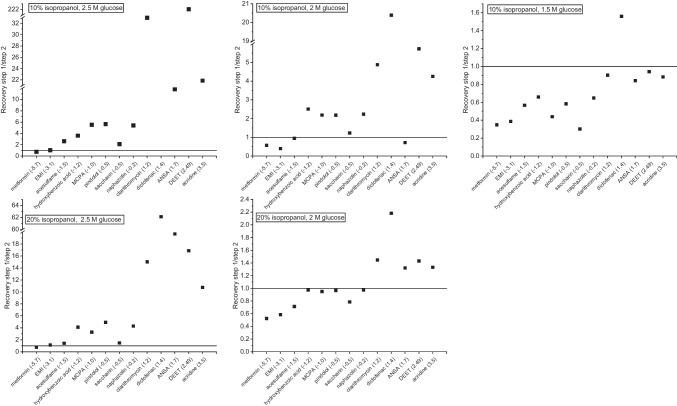
Recovery ratios rec(step 1)/(step 2) for all 13 model analytes and all tested extraction mixtures calculated from recoveries of SWIEET double extractions (see “Extraction procedure” section) from water with varying isopropanol content in the organic mixture (10–20%) and glucose concentration in the aqueous mixture (1.5–2.5 M)

In order to visualize the effects of the double extraction on the recovery of different analytes depending on their polarity, we calculated the recovery ratio rec(step1)/rec(step2) between the two extraction steps for each analyte and for different compositions of the extraction medium. Values <1 indicate a higher recovery in the second organic phase, while values >1 indicate a higher recovery in the first organic phase. Figure 2 shows that for all media, the second extraction step was more relevant to enhance the recoveries for polar analytes and especially for charged analytes, whereas nonpolar analytes were already well extracted in the first step. With 2.5 M glucose concentration, however, all ratios are >1 (except for metformin), demonstrating that the second step was not so efficient in enhancing the overall recoveries, which is especially the case for the least polar analytes with ratios >10. In contrast, using 1.5 M and 2 M glucose, for polar analytes (logD_pH7_ <0) ratios down to 0.2 were observed. For the final method chosen, the ratios were better balanced and remained in a range of 0.4 to 2.3 for all analytes. Values <1 for polar to charged analytes, values around 1 for analytes of intermediate polarity, and values >1 for nonpolar analytes were observed, leading to similar overall recoveries for all analytes with 90% on average. A QuEChERS extraction (see the “Extraction procedure” section) was conducted for comparison (Fig. 1k), which yielded 35% analyte recovery on average. This percentage was the lowest of the tested combinations for the first extraction step and was similar to SWIEET with 10% isopropanol and 1.5 M glucose as additives.

### Influence of glucose and isopropanol content on phase ratios in SWIEET

**Fig. 3:**
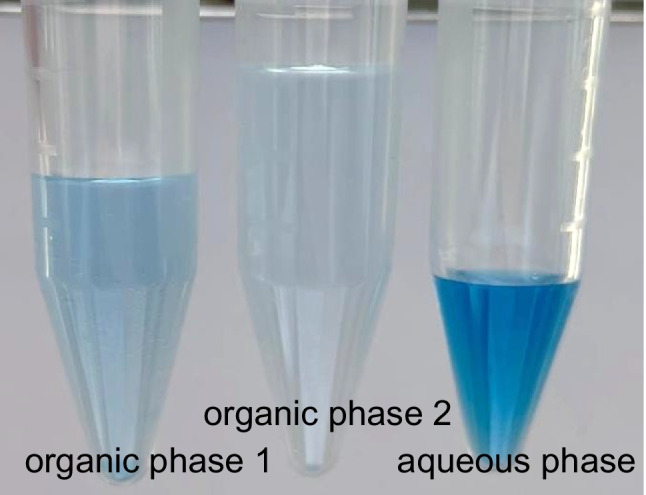
Photograph of the organic phases 1 and 2 from the SWIEET double extraction and the aqueous phase (right) after SWIEET double extraction from water with 2 M glucose and an organic mixture with 80–20 acetonitrile-isopropanol. Indigo carmine was added to the extraction mixture to visualize the water content. For the extraction procedure, see “Extraction procedure” section

In SWIEET extractions, recoveries for polar analytes were especially high in the second extraction step, when an organic mixture of 80% acetonitrile and 20% isopropanol was used for extraction. To better understand the reasons for this increase, we investigated the phase composition in SWIEET extractions. We presumed that a higher water content in the organic phase increases the polarity of the organic phase [31] and therefore the recoveries, especially of polar analytes. A higher water content was also expected to increase the recoveries of polar and charged analytes by a better solvation through hydrogen bonding. The water content was first visualized by adding indigo carmine. Indigo carmine is only soluble in water and solvent mixtures with water [3]. The water content can thus be estimated by the intensity of the blue color. As shown in Fig. 3, the first organic phase from SWIEET extraction has a deeper blue color than the second organic phase, indicating a higher water content in the organic phase during the first extraction. As expected, most of the indigo carmine remained in the aqueous phase.

Looking at the phase volumes for the different glucose and isopropanol concentrations (Fig. 1b, d, f, h, j), it is apparent that they were all very similar, with about 2.5 mL organic phase in the first extraction and 3 mL in the second step, except when using 1.5 M glucose and 10% isopropanol, where only a small volume of the first organic phase (2 mL) formed in the first step compared to the other extractions, while the volume of the second organic phase was significantly larger (3.5 mL). The combination of using 2 M glucose and 20% isopropanol for extraction also exhibited a notably larger organic phase volume in the second extraction step (3.5 mL). With QuEChERS (Fig. 1l), the phase volume was the same as in most of the first SWIEET extraction steps, with 2.5 mL in the organic and aqueous phases.

Using the intensity of the blue color from indigo carmine, a slight decrease in the water content in the second step vs. the first step was observed at all concentrations except 2.5 M glucose and 10% isopropanol, where almost no indigo carmine was visible in the organic phase in both steps. For extractions with 10% isopropanol and 1.5 M glucose (Fig. 1b), there was a similar intensity of the blue color in both phases in the first extraction step, indicating a similar water content in the organic and aqueous phases. In contrast, in the second step, a clear difference in the water content was observed. Using 20% isopropanol and 2 M glucose, a similar color was visible after both extraction steps; see Fig. 1h. For this combination, the highest water content of all experiments was reached in the second step. For QuEChERS, the water content was comparable to the first SWIEET extractions with 10% isopropanol and 2 M glucose or 20% isopropanol and 2.5 M.

Clearly, the higher water content correlated well with higher recoveries for different extraction media, as expected from an increase in the polarity of the organic phase. For metformin, 48% was recovered in the second step of the extraction with 20% isopropanol and 2 M glucose, where the highest water content was observed. At 10% isopropanol and 2.5 M glucose, where the water content was lowest in the second step, only 17% metformin was recovered. Especially, the combination of 20% isopropanol and 2 M glucose proved interesting, and further detailed experiments were conducted with this combination.

To directly investigate whether the sugar content has an influence on the phase ratio, the phases of two extractions were evaporated to reveal the sugar content in the organic and aqueous phases. We chose the final SWIEET additive composition (20% isopropanol, 2 M glucose) and, for comparison, a composition evoking low recoveries (10% isopropanol, 2.5 M glucose). The results are reported in Table 1. In the extraction with 10% isopropanol and 2.5 M glucose, only 4% of the glucose partitioned into the organic phase in the first and second extraction steps. The extraction with 20% isopropanol and 2 M glucose has a higher concentration ratio of 0.2 in the first and 0.09 in the second step. However, it is interesting to note that the higher initial glucose concentration of 2.5 M (with 10% isopropanol) compared to 2 M (with 20% isopropanol) does not lead to a higher glucose concentration in the organic phase. In addition, the phase ratio does not correlate with the glucose concentration in the organic phase; e.g., a concentration of 0.31 mol/L was determined in step 1 vs. 0.18 mol/L in step 2, where the phase ratio is larger.

**Table 1:** Glucose masses, estimated concentrations, and ratios between the organic and aqueous phases from both extraction steps of SWIEET double extractions with 10% isopropanol-2.5 M glucose and 20% isopropanol-2 M glucose (see “Extraction procedure” section and “Determination of the water, isopropanol, and glucose content” section)

			Glucose mass in mg	Estimated glucose concentration in mol/L	Glucose concentration ratio *c*(glucose_org_)/*c*(glucose_aq_)
10% isopropanol, 2.5 M glucose	Extr.1	Org. 1	8.8	0.097	0.04
		Aq. 1	209.1	2.3	
	Extr.2	Org. 2	9.1	0.093	0.04
		Aq. 2	175.4	2.1	
20% isopropanol, 2 M glucose	Extr.1	Org. 1	28.2	0.31	0.2
		Aq. 1	143.8	1.6	
	Extr.2	Org. 2	22.8	0.18	0.09
		Aq. 2	114.1	2.1	

### Determination of the water and isopropanol content

To better understand the composition of the phases, NMR measurements were conducted with the organic and aqueous phases of SWIEET and of QuEChERS. By the shift of the water signal in reference to the CD_3_CN signal in the ^1^H-NMR, the water content can be estimated in the organic phase [4]. We recorded NMR spectra of samples with known water content for calibration to estimate the water content in the samples (Table 2). For the first organic phase, the water signal was detected at 3.67 ppm on average (*n*=4); for the second organic phase, it was detected at 3.59 ppm on average, which was equivalent to water contents of 24% and 20%. This confirms the lower water content in the second organic phase, determined from visual inspection after the addition of indigo carmine. The organic phase of a QuEChERS extraction had the lowest water content, with an average shift of only 3.33 ppm, which was equivalent to a fraction of water of only 14%. For a more precise investigation of the water content, Karl Fischer titrations of the three organic phases were conducted, confirming the estimations made with NMR: the water content was 30.4 wt-% in the first SWIEET organic phase, 22.4 wt-% in the second SWIEET organic phase, and 10.9 wt-% in the QuEChERS organic phase. For all polar analytes (logD < 0), recoveries were higher in the second extraction step in SWIEET than in the first (e.g., metformin extr. 1: 25% vs extr. 2: 48%), although the water content was lower. Water content, therefore, is not sufficient to explain the increased recoveries in the second SWIEET extraction step compared to the first.

**Table 2:** NMR shifts of the water signal and isopropanol concentrations in the organic phases of SWIEET and QuEChERS extractions (see “Extraction procedure” section) determined using NMR (see “Determination of the water, isopropanol, and glucose content” section)

	Average water signal shift in ppm (*n*=4)	Water content estimated from NMR in %	Water content from Karl Fischer titration in %	Average isopropanol concentration in mol/L (*n*=4)
SWIEET organic phase 1	3.67 ± 0.01	24	30.4	0.82 ± 0.02
SWIEET organic phase 2	3.59 ± 0.02	20	22.4	1.02 ± 0.02
SWIEET aqueous phase 1	--	--	--	0.48 ± 0.02
SWIEET aqueous phase 2	--	--	--	0.40 ± 0.01
QuEChERS	3.33 ± 0.17	14	10.9	--

From the NMR measurements, we were also able to estimate the isopropanol content via the addition of maleic acid as an internal standard [32]. The isopropanol content proved to be higher in the second organic phase (1.02 mol/L on average) than in the first (0.82 mol/L on average). Isopropanol and acetonitrile contents were also estimated in the aqueous phases. The second aqueous phase contained a lower concentration of acetonitrile and isopropanol (1.52 mol/L and 0.40 mol/L) than the first (2.34 mol/L and 0.48 mol/L). Due to the ability to form H-bonds, isopropanol can aid in the solvation and, therefore, extraction of polar analytes. This was further investigated via solvatochromic parameters.

### Determination of solvatochromic parameters

For a deeper understanding of the influence of the phase properties on analyte extractions, solvatochromic parameters were determined. To calculate the solvatochromic parameters according to Kamlet and Taft [33], UV-Vis spectra of Reichardt’s dye, 4-nitroanisole, and 4-nitrophenol dissolved in the organic phases resulting from QuEChERS and SWIEET extraction with 2 M glucose and 20% isopropanol were recorded. For a better comparison, we performed an additional modified QuEChERS extraction with the same 80–20 acetonitrile-isopropanol mixture as used for SWIEET extractions. From the wavelengths of the absorption maxima, the parameters *α*, *β*, and π* were calculated using Eqs. (2–4) as described by Sindreu et al. [34] and Gonzalez-Arjona et al. [10]. The parameters for the extraction phases and mixtures of acetonitrile-water-isopropanol are summarized in Table 3.

**Table 3:** Average wavelengths *λ*_max_ (*n*=3, *n=1) of the dyes and resulting Kamlet-Taft parameters and E_T_(30) values determined in SWIEET and QuEChERS organic phases, as well as in known mixtures A to O with different fractions of isopropanol, water, and acetonitrile (remainder to 100%), given as the percentages of these three solvents. For details, see “Determination of solvatochromic parameters using UV-Vis”. *RD*, Reichardt’s dye; *4-NP*, 4-nitrophenol; *4-NA*, 4-nitroanisole

	*λ*_max_ (RD) in nm	*λ*_max_ (4-NP) in nm	*λ*_max_ (4-NA) in nm	*α*	*β*	π*	E_T_(30) in kcal/mol
QuEChERS	528 ± 5.4	313 ± 0.5	310 ± 0	0.76 ± 0.04	0.56 ± 0.02	0.79 ± 0	54.2 ± 0.6
SWIEET organic phase 1	516 ± 1.5	316 ± 1	311 ± 0	0.80 ± 0.01	0.62 ± 0.04	0.84 ± 0	55.4 ± 0.16
SWIEET organic phase 2	521 ± 1	315 ± 1	311 ± 0.6	0.79 ± 0.02	0.63 ± 0.04	0.82 ± 0.03	54.9 ± 0.1
SWIEET combined	519 ± 0	315 ± 1	311 ± 0.5	0.80 ± 0.02	0.62 ± 0.06	0.83 ± 0	55.1 ± 0
QuEChERS with 20% isopropanol	510 ± 3	315 ± 0	311 ± 0	0.84 ± 0.03	0.60 ± 0	0.84 ± 0	56.0 ± 0.33
80–20 acetonitrile-isopropanol	576 ± 1.7	312 ± 0	308 ± 0	0.58 ± 0.01	0.59 ± 0	0.71 ± 0	49.6 ± 0.15
SWIEET aqueous phase 1	--	317 ± 0	315 ± 1.5	--	--	1.03 ± 0.07	--
SWIEET aqueous phase 2	--	318 ± 0	315 ± 0	--	--	1.01 ± 0	--
Pure acetonitrile	626 ± 2.9	307 ± 1	308 ± 0	0.35 ± 0	0.34 ± 0.03	0.70 ± 0	45.6 ± 0.21
Pure isopropanol	592 ± 1.5	312 ± 0	304 ± 0	0.63 ± 0.01	0.76 ± 0	0.52 ± 0	48.3 ± 0.12
Mix A*: 5% iPr, 20% H_2_O	523	314	310	0.79	0.60	0.79	54.7
Mix B*: 10% iPr, 20% H_2_O	523	314	310	0.79	0.60	0.79	54.7
Mix C*: 15% iPr, 20% H_2_O	525	315	310	0.78	0.64	0.79	54.5
Mix D*: 5% iPr, 15% H_2_O	529	314	310	0.76	0.60	0.79	54.0
Mix E*: 10% iPr, 15% H_2_O	531	314	310	0.75	0.60	0.79	53.8
Mix F*: 15% iPr, 15% H_2_O	532	314	310	0.74	0.60	0.79	53.7
Mix G*: 5% iPr, 25% H_2_O	520	315	310	0.81	0.64	0.79	55.0
Mix H*: 10% iPr, 25% H_2_O	520	315	311	0.78	0.60	0.84	55.0
Mix I*: 15% iPr, 25% H_2_O	522	315	310	0.80	0.64	0.79	54.8
Mix J*: 5% iPr, 30% H_2_O	516	315	311	0.81	0.60	0.84	55.4
Mix K*: 10% iPr, 30% H_2_O	520	315	311	0.78	0.60	0.84	55.0
Mix L*: 15% iPr, 30% H_2_O	524	316	312	0.73	0.60	0.88	54.6
Mix M*: 5% iPr, 35% H_2_O	514	315	312	0.79	0.56	0.88	55.6
Mix N*: 10% iPr, 35% H_2_O	514	315	311	0.82	0.60	0.84	55.6
Mix O*: 15% iPr, 35% H_2_O	517	316	312	0.77	0.60	0.88	55.3

To extend the interpretation of the solvatochromic parameters of our solvent mixtures, we conducted further measurements with known mixtures of isopropanol, acetonitrile, and water. Isopropanol and water contents were in the range of the values determined in the “Determination of the water and isopropanol content” section. The resulting wavelengths and parameters are also listed in Table 3.

**Fig. 4:**
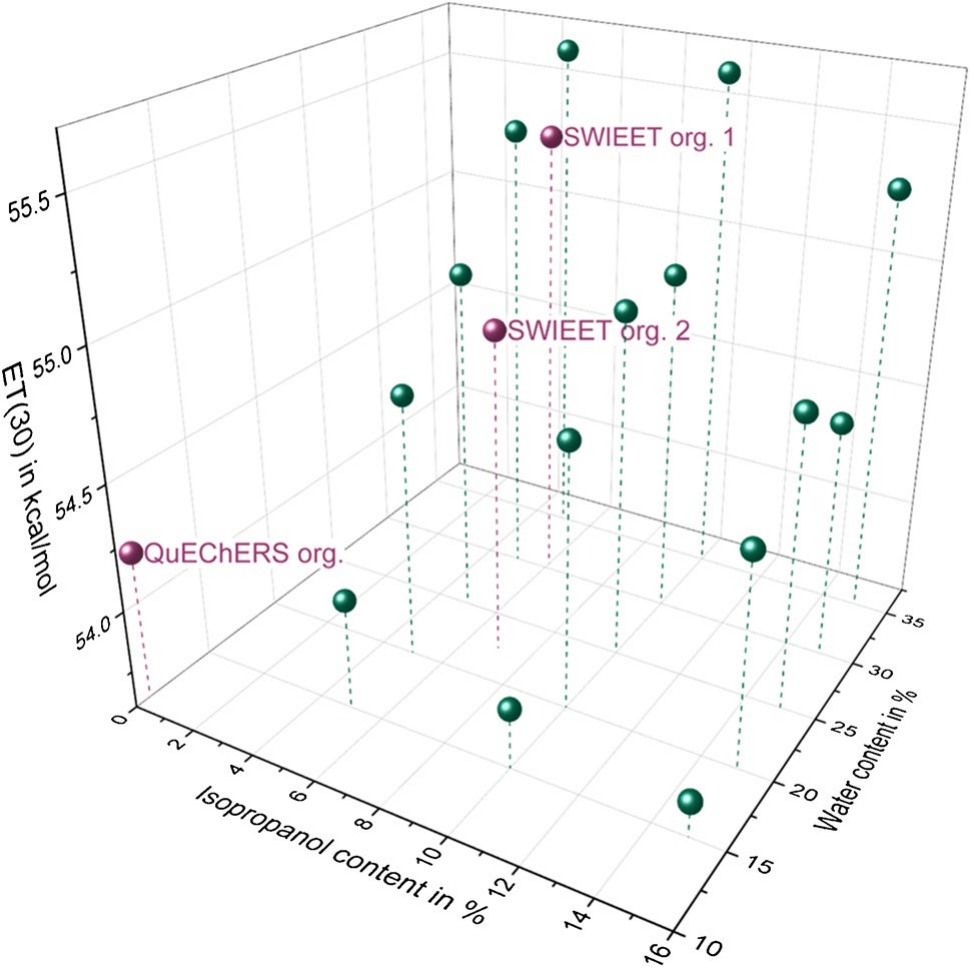
E_T_(30) values for mixtures A–O of isopropanol and water in acetonitrile listed in Table 3, as well as values for SWIEET org. 1, SWIEET org. 2*,* and QuEChERS org. For details on the determination of the E_T_(30) value, see “Determination of solvatochromic parameters using UV-Vis section”

The E_T_(30) value determined via the addition of Reichardt’s dye is a measure of the overall polarity of a solvent mixture. The E_T_(30) parameter is very sensitive to polarity changes, given the large shifts of the absorption maxima of Reichardt’s dye upon changes in the solvent composition. The overview of E_T_(30) values in Fig. 4 shows that, with increasing isopropanol content in the mixture, E_T_(30) values decreased, whereas, with increasing water content, E_T_(30) values increased. Comparing the slopes of the dependencies, it is obvious that the water content influenced the E_T_(30) parameter more strongly than isopropanol. This is also in line with the results from an analysis of variance of recoveries of individual analytes depending on glucose and isopropanol concentrations: Here, we found that varying isopropanol concentration yielded fewer significant differences in recoveries for single analytes than varying glucose concentrations. The E_T_(30) values of our known mixtures varied between 53.7 and 55.6 kcal/mol and were between those for pure acetonitrile (45.6 kcal/mol, aprotic), isopropanol (48.4 kcal/mol, protic), and water (63.1 kcal/mol, protic) listed by Reichardt [5] as expected for mixtures. Of our unknown solvent mixtures from extractions, the E_T_(30) values of all mixtures except modified QuEChERS with isopropanol (56 kcal/mol) lay in the range of the known solvent mixtures. The mixture of 80–20 acetonitrile-isopropanol, which was added for SWIEET extraction, had an E_T_(30) value of 49.6 kcal/mol, which is higher than the neat solvents. E_T_(30) values were lower for the organic phase in QuEChERS (54.2 kcal/mol) compared to SWIEET (54.9–55.9 kcal/mol). As shown in Fig. 4, even small variations of the mixture composition led to large differences in the E_T_(30) parameter, especially when changing in the water content.

The Kamlet-Taft parameter *α* is a measure for the hydrogen bond–donating capacity of the solvent compared to methanol used as a reference. A similar trend as for the E_T_(30) values was observed along the solvent mixtures for *α*: a lower isopropanol content and higher water content increased *α* (the absorption maxima of Reichardt’s dye are included in the calculation of *α*). The values for *α* varied between 0.74 and 0.81 for the known mixtures A–O. For the extraction phases tested, the organic phase of QuEChERS had the lowest value of *α* with 0.76, meaning that it has a weaker hydrogen bond–donating capacity than the SWIEET organic phases ranging from 0.79 to 0.80. The modified QuEChERS extraction with isopropanol had the highest *α* value of 0.84 and was outside the values determined for the known solvent mixtures. Kamlet and Taft reported average *α* values of 0.69 for isopropanol, 0.29 for acetonitrile, and 1.02 for water [7]. To obtain these values, they averaged the values resulting from six different calculation methods, based on different solvatochromic probes. For a better comparison, we determined the *α* values also in this study as described above. They were 0.35 for acetonitrile and 0.63 for isopropanol and differed slightly from the literature values. For comparison, we also determined the parameter *α* for the 80–20 acetonitrile-isopropanol mixture used for extraction. The *α* value was between the neat solvents (0.58) as can be expected. The parameter for water could not be determined since the solubility of Reichardt’s dye was insufficient.

The parameter β, which describes the hydrogen bond–accepting properties of the solvent relative to DMSO, was lowest for the QuEChERS organic phase (0.56) compared to SWIEET (0.62–0.63). The isopropanol-QuEChERS organic phase revealed an absorption maximum between QuEChERS and SWIEET, which resulted in *β*=0.60. For the neat solvents isopropanol, water, and acetonitrile, literature values for the parameter *β* vary greatly [33, 35]: For example, for water, Kamlet et al. [33] listed an average of *β*=0.13 for water, acquired with different probes, while Marcus [35] listed *β*=0.47. For isopropanol, Kamlet and Taft reported *β* values in the range of 0.91 to 0.95 [8] and Marcus *β*=0.84, for acetonitrile; Marcus listed *β*=0.4 [35]. In our experiments, we calculated *β* values of 0.76 and 0.34 for isopropanol and acetonitrile, respectively, and 0.59 for the 80–20 mixture. Compared to the differences in the values of these parameters between neat solvents, the values of the organic mixtures tested in QuEChERS and SWIEET can be regarded as being similar in the two methods.

For the parameter π*, a measure for polarizability and dipolarity [5], similar values of 0.82–0.84 for SWIEET, 0.79 for QuEChERS, and 0.84 for isopropanol-QuEChERS were obtained. The calculation of *β* and π* is based on the absorption maxima of 4-nitrophenol and 4-nitroanisole, which are not as sensitive to changes in the solvent composition as Reichardt’s dye. Sindreu et al. [34] determined Kamlet-Taft parameters for aqueous mixtures of tert-butanol and ethylene glycol. Their values varied depending on the ratio of the solvents: For π* and *α*, they differed by up to 0.6; for *β* up to 0.24. In the work of Cheong et al. [36], the values for π* of water-cosolvent mixtures differed by up to 0.67, depending on the volume fraction of the cosolvent. For example, an increase in the volume fraction of methanol, acetonitrile, isopropanol, or tetrahydrofuran by 10% changed π* by 0.01–0.15. In our experiments, π* of the QuEChERS organic phase (0.79) and the SWIEET organic phase (0.82–0.84) differed by 0.05. The value for isopropanol-QuEChERS was identical to SWIEET organic phase 1 (0.84). The π* value for the 80–20 acetonitrile-isopropanol mixture was higher compared to the one for the neat solvents, as opposed to *α* and *β*. The differences in beta values for the extraction phases were not regarded significant compared to the range of values for the mixtures of known composition.

To further broaden the understanding of the extractions, we also investigated the aqueous phases of the extraction methods. The solubility of Reichardt’s dye was too low to reach a sufficient concentration to record UV/Vis spectra. Thus, only the parameter π* could be determined for SWIEET. For QuEChERS, no absorption maxima were detected for 4-nitrophenol and 4-nitroanisole, possibly due to the low solubility of the dyes in water. The parameter π* of the first and second aqueous SWIEET phases did not differ significantly (1.03 vs. 1.01). However, the elevated organic content revealed a π* value significantly different from that of pure water of 1.09 [9] in both steps.

All Kamlet-Taft parameters of the organic solvent mixtures determined lay between the literature values for the neat solvents. Comparing the SWIEET organic phases, the differences for the Kamlet-Taft parameters were within the standard deviation, so no significant differences were present. The largest differences were observed between QuEChERS and SWIEET, and isopropanol-QuEChERS and SWIEET for all parameters.

## Discussion

In this study, we compared the phase composition and Kamlet-Taft parameters for organic phases in QuEChERS and SWIEET. In addition, we investigated different extraction media in SWIEET double extractions [3] varying glucose and isopropanol content to understand their relevance for analyte recoveries. For promising extraction media, the water content of the organic phases of both extraction steps was determined by Karl Fischer titration, and the isopropanol content was determined by NMR spectroscopy to gain insight into the differences in the phase composition in the two extraction steps, with their different recoveries, especially for polar and charged analytes [3]. Furthermore, solvatochromic parameters of the phases are determined to provide information about polarity and specific solvent-solute interactions and are compared to the parameters of known mixtures.

The experiments with indigo carmine indicated a lower water content in the second organic phase in the SWIEET double extraction. For QuEChERS extracts, the water content was the lowest of all experiments. This was confirmed by NMR and Karl Fischer titration experiments, the latter with precise numbers [37]. These findings are in line with the lower values of the π* parameter in the second aqueous phase compared to the first. Comparing SWIEET extraction steps, no direct correlation of the recovery for single analytes, analyte groups (polar and nonpolar), or the overall average recovery to the water content was observed. In contrast, an elevated water content might play a role regarding the increased recovery with SWIEET extractions compared to QuEChERS extractions, but the database is not yet sufficient for a clear explanation. The recovery ratios of the first and second extraction steps for single analytes with different extraction media show that isopropanol and glucose concentrations can be adjusted regarding the polarity of the target analyte, with extraction media evoking higher phase ratios being of interest, especially for polar analytes, whereas for nonpolar analytes, high glucose and lower isopropanol content seem advantageous and may allow the omission of the second extraction step. The small ratios present for many analytes with 20% isopropanol and 2 M glucose and even more for 10% isopropanol and 1.5 M glucose point to the relevance of the phase ratio in the second extraction step.

We regard the water content not to be sufficient as a sole reason for explaining the increased recoveries for polar analytes in SWIEET compared to QuEChERS. We therefore hypothesize that, besides the difference in the water content, the addition of the protic solvent isopropanol enhanced the solubilization and thus recoveries of polar and charged analytes, especially when the water content was relatively low. This is due to the ability of isopropanol to form hydrogen bonds in contrast to the aprotic acetonitrile. The increased isopropanol content in the second organic phase supports this hypothesis. As a measure to quantify the hydrogen bond–donating and hydrogen bond–accepting capacities of the organic phase, Kamlet-Taft parameters were determined. Although the organic mixtures added to QuEChERS and SWIEET both mainly consist of acetonitrile (100% and 80%), the resulting organic phases exhibited different hydrogen bond–donating and hydrogen bond–accepting capabilities. In SWIEET, isopropanol is added to the organic extraction mixture, which increases the hydrogen bond–donating and hydrogen bond–accepting capabilities of the resulting organic phase after phase separation, beside the effects exerted by the water content. However, the parameters *α* and *β* for the organic phases of both SWIEET extraction steps and QuEChERS did not differ significantly, and no correlation to analyte recoveries was seen.

In contrast, strong differences between QuEChERS and SWIEET were observed in the parameter E_T_(30), as well as between the two extraction steps in SWIEET double extraction. Using Reichardt’s dye to determine E_T_(30) proved that the overall polarity was lower in the QuEChERS organic phase than in the two SWIEET organic phases. In addition, the overall polarity was highest in the first organic phase and slightly lower in the second in SWIEET. The higher isopropanol content did not significantly influence the Kamlet-Taft parameters. The value for the combined SWIEET organic phases was between the ones for the separate first and second, which is expected since it is a mixture of both. All mixtures of acetonitrile and water (plus isopropanol in case of SWIEET) investigated yielded higher E_T_(30) values than neat acetonitrile and would be classified as protic solvents (47–63 kcal/mol). Langhals [31] observed that a small amount of a polar solvent added to a nonpolar solvent has an unproportionally strong effect on the polarity of the mixture. E_T_(30) measurements of mixtures in the literature also revealed that the parameter is not always linearly related to the solvent ratio due to selective solvation of the dye [5]. In ternary mixtures, this relation is even more complex, and antagonistic effects may be present. This might explain why the three different SWIEET organic phases (the combined and two separate phases) showed relatively similar E_T_(30) values despite their significantly different composition, as determined by NMR and Karl Fischer titration.

Comparing the extractions with different additive compositions, it seems that higher initial glucose concentrations are not automatically linked to higher contents of glucose in the organic phase, as with 2.5 M glucose in the extraction medium (10% isopropanol) the glucose concentration in the organic phase were 97 and 93 mM, whereas in the combination of 2 M glucose with 20% isopropanol, we estimated concentrations of 310 and 180 mM of glucose in the first and second step. For glucose concentrations of 2 M, the isopropanol content caused significant differences (ANOVA/*t*-test) especially for polar and charged analytes like metformin, EMI, and acesulfame. These analytes profited most from a higher isopropanol content due to its ability to form H-bonds. It thus seems that the isopropanol content is decisive for the amount of glucose present in the organic phase, but not so much the amount of glucose for the isopropanol content. This would mean that the polar glucose, just like polar analytes, is better extracted at higher isopropanol content, but does not define the extractability of the analytes.

To better judge the differences in the E_T_(30) values, we established a scale for various ternary mixtures of the three solvents isopropanol, acetonitrile, and water over a relatively wide range of compositions (see Table 3). E_T_(30) values covered a range of only 53.7 to 55.6. All organic phases revealed values within this range (SWIEET 1 and 2: 55.4 and 54.9 kcal/mol vs. QuEChERS 54.2 kcal/mol). Interestingly, the value for QuEChERS strongly increased to 56.0 kcal/mol when adding 20% isopropanol to the organic mixture prior to extraction, indicating a relatively strong effect of isopropanol. From the mixtures of the solvents A to O (see Table 3), a clear increase of the E_T_(30) parameter upon a decreasing isopropanol and increasing water content was observed (see Fig. 4). This trend was also visible when comparing the organic phases after extraction: From Karl Fischer titration, we know the water content of the organic phases of SWIEET (organic phase 1: 30.4%, organic phase 2: 22.4%) and QuEChERS (10.9%), as well as the isopropanol content from NMR (organic phase 1: 6%, organic phase 2: 8%), which is in line with the trend for the parameter E_T_(30), albeit no linear correlation was observed.

This may be due to effects by the salts used in QuEChERS: For QuEChERS, the E_T_(30) parameter of the organic phase after extraction increased by 8.6 kcal/mol compared to neat acetonitrile. Similarly, a strong increase of 6.4 kcal/mol is seen between 80–20 acetonitrile-isopropanol and the QuEChERS organic phase with isopropanol. This might not only be due to the water content, but also halochromism: During QuEChERS extraction, salts are added to induce phase separation, which will partly partition into the organic phase. Reichardt’s dye was reported to be cationic halochromic, meaning its absorption maximum is sensitive, especially to cations in an electrolyte, and a hypsochromic shift (blue shift, higher excitation energy) can be observed [6]. This shift was shown to be more intense after the addition of cations with high charge density [6], such as Mg^2+^, which is also used in QuEChERS extractions. Summarizing, an increase in polarity/polarizability seems to be relevant for the differences between SWIEET and QuEChERS, but not for the differences between the steps in SWIEET double extractions.

Overall, neither water content, nor isopropanol content, nor glucose concentration, nor the polarity of the organic phases was able to satisfactorily explain the increased recoveries in the second step in SWIEET double extractions at 20% isopropanol and 2 M glucose, compared to the first step. The high recoveries of analytes obtained using this combination seem to be dominantly based on the very high phase ratio of the organic to aqueous phase in the second SWIEET extraction step, in combination with a higher similarity between the organic and aqueous phases indicated by all parameters investigated. The increased phase volume of the second organic phase can be explained by the principle of the lever in miscibility gaps: Due to a fraction of water being removed with the organic phase in the first step and only organic solvent being added for the second extraction, the amount of water available during phase separation in the second step is lower than in the first. This shift in the overall composition towards organic solvents lengthens the lever arm in the phase diagram to the phase rich in organics, and thus its volume increases, and thus also the phase ratio. The phase ratio directly influences recoveries, as shown in [3]. Clearly, the second organic phase must contain a higher fraction (but not concentration) of the total amount of water than the first, since the aqueous phase volume decreases from 2.5 to 1.5 during the second step and the organic phase volume increases to 3.5 mL, beyond the volume of extraction medium added (2.5 mL), see Fig. 1h. We presume that this shift in the phase ratio is crucial to improve the recoveries in the double extraction.

A reason why this effect on the phase ratio is only observed at the highest isopropanol content may lie in the structure of isopropanol-water clusters: When the isopropanol content in water is increased, a change in the hydrogen bonding network is observed at a mole fraction of 0.2 isopropanol, and the conformation of the isopropanol-water clusters changes [38]. Guo et al. showed that isopropanol clusters with five water molecules form the most stable hydrogen bonds [39]. In the second SWIEET extraction step, the addition of organic mixtures led to an increased isopropanol content of 6 vs. 8% as determined by NMR. This might pull water into the organic phase to result in more stable isopropanol-water clusters. A similarly intense shift in phase volume was not observed at glucose concentrations higher than 2 M. It can be anticipated that an increased glucose concentration leads to a competition with isopropanol for the water molecules, as they are required for solubilization, based on an excluded volume effect [40]. At high glucose concentrations in water, clustering of glucose molecules was observed on a microscopic level [41]. Glucose-glucose interactions increase when water-water interactions decrease; therefore, less water will be available for the solvation of isopropanol in the organic phase. As we described previously [3], the permittivity decreases in higher concentrated glucose solutions [42], which also aids extraction of polar analytes due to a decreased solubility in the aqueous phase. A further increase of the isopropanol content is hypothesized to also increase recoveries, but would result in unstable phase separations due to increased miscibility. Even if a phase separation may be reached, robustness will be lowered, especially in the case of samples with a higher matrix load. Similarly, at glucose concentrations lower than 1 M, no stable phase separation was obtained with isopropanol present. Thus, a combination of 2 M glucose and 20% isopropanol proved best for extractions in SWIEET.

To conclude, we found that a complex counterplay between phase composition and phase ratio is decisive for the recoveries achievable in liquid-liquid extractions. For the difference in recoveries of polar analytes in SWIEET vs. QuEChERS, the water content and the addition of isopropanol were significant. The difference in recoveries between the first and second organic phase of SWIEET double extractions can be explained by the higher phase ratio present in the second step. This understanding can be used to tune the SWIEET method parameters to fit specific applications if a specific group of analytes is of interest: Depending on the sample type and target analytes characteristics, phase polarity and ratio can be optimized by the amount of glucose and isopropanol added to improve recoveries and separation from matrix components. High isopropanol concentration can be expected to aid in the extraction of highly polar and also charged analytes, whereas a high glucose concentration will aid in reaching high extraction efficiencies for less polar analytes. A major conclusion from this fundamental study is that SWIEET is better suited for analytes solubilized via hydrogen bonding, such as charged analytes, than QuEChERS.

## Data Availability

All necessary data are included in the paper. Should any raw data files be needed, they are available from the corresponding author upon reasonable request.
